# A developmental atlas of male terminalia across twelve species of *Drosophila*

**DOI:** 10.3389/fcell.2024.1349275

**Published:** 2024-02-29

**Authors:** Anna Urum, Gavin Rice, William Glassford, Yifat Yanku, Boris Shklyar, Mark Rebeiz, Ella Preger-Ben Noon

**Affiliations:** ^1^ Department of Genetics and Developmental Biology, The Rappaport Faculty of Medicine and Research Institute, Technion - Israel Institute of Technology, Haifa, Israel; ^2^ Department of Biological Sciences, University of Pittsburgh, Pittsburgh, PA, United States

**Keywords:** male genitalia, *Drosophila*, evolution, morphogenesis, pupal terminalia

## Abstract

How complex morphologies evolve is one of the central questions in evolutionary biology. Observing the morphogenetic events that occur during development provides a unique perspective on the origins and diversification of morphological novelty. One can trace the tissue of origin, emergence, and even regression of structures to resolve murky homology relationships between species. Here, we trace the developmental events that shape some of the most diverse organs in the animal kingdom—the male terminalia (genitalia and analia) of *Drosophilids*. Male genitalia are known for their rapid evolution with closely related species of the *Drosophila* genus demonstrating vast variation in their reproductive morphology. We used confocal microscopy to monitor terminalia development during metamorphosis in twelve related species of *Drosophila*. From this comprehensive dataset, we propose a new staging scheme for pupal terminalia development based on shared developmental landmarks, which allows one to align developmental time points between species. We were able to trace the origin of different substructures, find new morphologies and suggest possible homology of certain substructures. Additionally, we demonstrate that posterior lobe is likely originated prior to the split between the *Drosophila melanogaster* and the *Drosophila yakuba* clade. Our dataset opens up many new directions of research and provides an entry point for future studies of the *Drosophila* male terminalia evolution and development.

## 1 Introduction

The evolution of morphology results from genetic changes that are manifested during development. Traditionally, evolutionary genetic studies have concentrated on establishing a causal link between genetic and phenotypic changes (Martin and Orgogozo, 2013; Courtier-Orgogozo, 2023). However, the developmental processes responsible for translating these genetic changes into novel morphologies often remain in the shadows. One significant obstacle exists for studying the development of novel traits that seem to appear out of thin air in the evolutionary record. For these traits, it is frequently difficult to identify species comparisons that are sufficiently close to infer homology but still display highly divergent morphology. The evolution of male genitalia in *Drosophila* presents a unique system to overcome these challenges as it provides a rare opportunity to uncover the developmental pathways and mechanisms responsible for shaping extremely diverse forms observed across closely related species.

**FIGURE 1:**
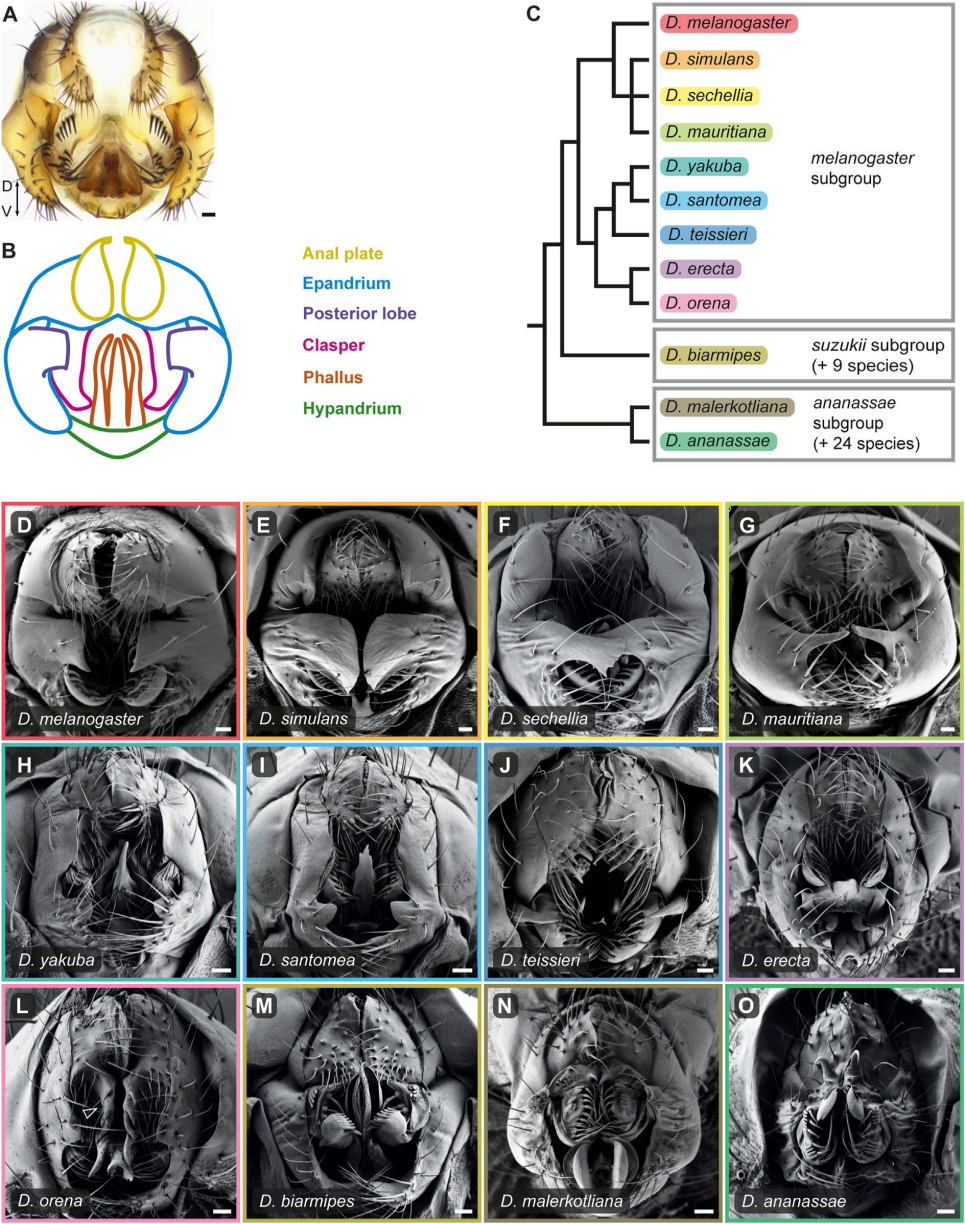
The male terminalia of *D. melanogaster* species group undergo rapid evolution. **(A)** Light microscopy image of *D. melanogaster* adult male terminalia. **(B)** Schematic representation of the major terminal substructures of adult *D. melanogaster*. The different substructures are color-coded according to the index on the right. Adapted from Vincent et al. (2019). **(C)** Phylogeny for twelve species of the *D. melanogaster* species group based on (Obbard et al., 2012). Boxes indicate subgroups within this species group. **(D–O)** Scanning electron micrographs of adult male terminalia of the twelve species presented in the phylogeny in **(C)**. The frame color of each panel corresponds to the color highlighting the species name in **(C)**. Arrowhead in (L) indicates the enlarged ventral cercal lobes of *D. orena*. Dorso-Ventral (D–V) axis direction is indicated in panel **(A)**. Scale bars: 20 µm.

Male genitalia are among the most diverse and rapidly evolving organs in the animal kingdom, with sexual selection as the most cited factor (Eberhard, 1985). This trend extends to the model organism *Drosophila melanogaster* and its close relatives, which display dramatic morphological differences posited to contribute to reproductive success (Kopp and True, 2002; Masly, 2012) (Figure 1). These striking differences in male genital morphologies have long captivated biologists, who used them as a model to study the genetic basis of morphological evolution (Coyne, 1983; True et al., 1997; Macdonald and Goldstein, 1999; Zeng et al., 2000; Masly et al., 2011; McNeil et al., 2011; Peluffo et al., 2015; Takahara and Takahashi, 2015; Tanaka et al., 2015; Hagen et al., 2019), evolutionary innovations (Kopp and True, 2002; Yassin and Orgogozo, 2013; Glassford et al., 2015; Smith et al., 2020), gene regulatory network (GRN) architecture and co-option (Glassford et al., 2015), and reproductive isolation (Kopp and True, 2002; Masly, 2012; Frazee et al., 2021). In addition, male genital morphologies are often the most reliable means to distinguish between closely related species of *Drosophila* visually and are therefore crucial for taxonomical classification (Bock and Wheeler, 1972). Thus, the striking diversity of *Drosophila* genitalia that has evolved over relatively short evolutionary distances poses unique challenges in determining homology relationships among structures that appear wildly different and the mechanisms that generate such morphological richness.

The adult terminalia (that include the genitalia and the analia) develop from the larval genital disc during metamorphosis through extensive cell proliferation and epithelial remodeling (Estrada et al., 2003; Glassford et al., 2015; Smith et al., 2020; Rice et al., 2023). We have recently traced the development of the phallus in eight members of the *D. melanogaster* species group (Rice et al., 2023). We discovered that adult phallic processes originate from three primordia and that in some instances, structurally similar phallic processes arise from the same primordia, while in other cases, apparently homologous processes develop from different primordia and are thus non-homologous (Rice et al., 2023). To date, the cellular processes involved in genital morphogenesis have been investigated for only two specialized genital structures. First, Smith et al. (2020), have shown that the posterior lobe, a copulatory structure unique to the *D. melanogaster* complex, arises through an extreme increase in epithelial cell height that is facilitated by interactions with the apical extracellular matrix (aECM) protein Dumpy (Smith et al., 2020). Second, Green et al. (2019) found that the enlarged ovipositor in females of *D. suzukii* develops through an accelerated expansion of the apical cell area combined with anisotropic cell rearrangements (Green et al., 2019). To date, there is little to no research on developmental differences in analia despite evidence of anatomical variation (Kopp and True, 2002). Much more work is needed to determine what other cellular behaviors participate in terminalia morphogenesis and diversification.

The genetic pathways that specify the *D. melanogaster* genital disc have been studied predominantly in the context of the larva, where several genes that control the fate of the adult structures were identified (Chen E.H. and Baker B.S., 1997; Keisman and Baker, 2001; Estrada et al., 2003; Chatterjee et al., 2011). Only a handful of studies focused on the genes and networks that pattern the genitalia during metamorphosis (Glassford et al., 2015; Hagen et al., 2019; 2021; Vincent et al., 2019; Smith et al., 2020; Ridgway et al., 2023). For example, Glassford et al. (2015) studied the origin of the posterior lobe and found that it emerged in the *D. melanogaster* clade through the co-option of an ancestral embryonic Hox-regulated GRN that controls the development of the larval posterior spiracle (Glassford et al., 2015). Hagen et al. (2019) used high-resolution genetic mapping to identify genes that are involved in clasper size differences between *D. simulans* and *D. mauritiana*. They found that variations in the expression levels of *tartan*, a gene that encodes a transmembrane protein involved in cell–cell interactions, contribute to clasper size differences between these species (Hagen et al., 2019)*.* Finally, to further our knowledge of GRNs participating in pupal terminalia development, Vincent et al. (2019) have created an online open database for gene expression patterns in the *D. melanogaster* terminalia (flyterminalia.pitt.edu). This database contains RNA *in situ* hybridization images for 100 transcription factors in male pupal terminalia at two developmental timepoints (Vincent et al., 2019). While these studies represent major advances toward uncovering the genes and pathways that regulate specific structures during male genitalia development in *D. melanogaster* and its closely related species, we are still missing a comprehensive description of pupal terminalia development in most of these species and in other species in more distantly related groups.

To gain insights into the developmental processes that diversify male genitalia and analia across evolution, we monitored pupal terminalia development in twelve *Drosophila* species using confocal microscopy. We uncovered multiple morphogenetic events that produce a wide variety of unique genital substructures. In addition, we demonstrate that the posterior lobe emerged in the *melanogaster* subgroup of species prior to the split between the *D. melanogaster* and *D. yakuba* complexes through shared developmental and molecular programs. Our dataset offers a much-needed foundation for researchers in the field to study diverse facets of genitalia development and evolution.

## 2 Materials and methods

### 2.1 *Drosophila* strains

The following stocks were obtained from the National *Drosophila* Species Stock Center at UCSD (now located at Cornell University): *D. santomea* (14021-0271.01), *D. teissieri* (14021-0257.01), *D. orena* (14021- 0245.01), *D. erecta* (14021-0224.01), *D. biarmipes* (14023-0361.09), *D. ananassae* (14024- 0371.13). *D. sechellia* (14021-0248.28), *D. melanogaster* OregonR, *D. simulans, D. mauritiana* and *D. yakuba* wild type strains were a kind gift from Dr. David Stern. *D. malerkotliana* was a kind gift from the lab of Dr. Thomas Williams.

### 2.2 Light microscopy imaging of the adult genitalia

Adult males were dissected in ethanol and their phallic structures were removed. The periphallic parts were placed on slides in glycerol mounting solution (80% Glycerol, 10% 1M Tris HCl pH 8.0) and imaged at ×20 and ×10 magnification on a Leica DM 2000 equipped with a Leica DFC450C camera.

### 2.3 Scanning electron microscopy imaging of the adult terminalia

Anesthetized adult males were transferred into 100% ethanol and kept at −20°C for 7 days. Ethanol was replaced every 2 days for dehydration. On the seventh day, the whole abdomens were dissected. After dehydration, the specimens were critical point dried (Quorum K850), and sputter coated with 8 nm of Iridium (Quorum Q150T). The samples were viewed using SE2 detector at accelerating voltage of 1 kV on Zeiss Ultra Plus HR Scanning Electron Microscope.

### 2.4 Confocal imaging of pupal terminalia

Flies were incubated at 25°C prior to collection. Male white pre-pupae were collected and aged to the appropriate developmental time point (measured in hours after puparium formation, or hAPF) at 25°C in a Petri dish containing a moistened Kimwipe. The formation of a white pre-pupae occurs over a 30–60 min interval, which introduces slight variations in timing from sample to sample (in addition to individual-to-individual differences in development). The posterior tip of the pupa (20%–40% of pupal length) was separated in PBS using micro-dissection spring scissors (Fine Science Tools #15000-04) and washed with PBS to flush out the pupal terminalia. Samples were fixed in 4% paraformaldehyde in PBT (PBS with 0.1% Triton-X-100) at room temperature for 30 min, and then washed 4 times with PBT. Fixed samples were maintained in PBT at 4°C for up to 2 weeks.

The fixed samples were stained with anti-E-cadherin (Huang et al., 2012) to visualize apical cell junctions. Briefly, the samples were incubated with rat anti-E-cadherin (DSHB Cat# DCAD2,RRID:AB_528120), 1:100 in PBT, or rabbit anti-Ems (Dalton et al., 1989), 1:200 in PBT, overnight at 4°C, washed several times with PBT and then incubated with donkey anti-rat Alexa 488, 1:200 (Thermo Fisher Scientific #A-21208), Cy™3-conjugated AffiniPure Goat Anti-Rat IgG (H + L) (Jackson ImmunoResearch, 112-165–167), 1:100, or donkey anti-rabbit Alexa 647 at 1:400 dilution (Molecular Probes) overnight at 4°C. The samples were mounted on slides covered with poly-L-lysine (Thermo Fisher Scientific #86010 and Sigma-Aldrich P4832), in glycerol mounting solution (80% Glycerol, 10% 1M Tris HCl pH 8.0) and imaged at 20X on Zeiss LSM 900 Airyscan 2 and Leica TCS SP8 confocal microscopes. The confocal images were processed in Imaris^©^ Bitplane AG, using the Surfaces visualization function to generate 3D models. At least three samples were analyzed for each data point. Images of pupal terminalia that were previously used in Rice et al. (2023) are summarized in Supplementary Table S1.

## 3 Results

### 3.1 *D. melanogaster* male terminalia anatomy and development

The adult male terminalia of *Drosophila* is a bilaterally symmetrical anatomical structure located at the posterior end of the adult male abdomen (segments 8–10). It can be subdivided into two parts: the phallic structures and the periphallic structures (Figure 1; Supplementary Figure S1). A standardized nomenclature for these structures has been previously established (Rice G. et al., 2019a) and any time we break from this standard, the technical term is provided in parentheses. The phallic structures include the phallus and the hypandrium and play important roles during copulation, including participation in genital coupling and sperm transfer. The periphallic structures consist of the anal plates (cerci), the genital arch (epandrium), a pair of claspers (surstyli), and the subepandrial sclerite that connects the claspers to the anal plates. The epandrium includes the epandrial dorsal lobes, the lateral plates (epandrial ventral lobes), and in species of the *D. melanogaster* complex the posterior lobes (epandrial posterior lobes) that protrude from the lateral plates. The periphallic structures form physical interactions with the female genitalia, facilitating genital coupling during copulation (Robertson, 1988; Kopp and True, 2002; Acebes et al., 2003; Jagadeeshan and Singh, 2006; Kamimura and Mitsumoto, 2011; Yassin and Orgogozo, 2013; Glassford et al., 2015; Mattei et al., 2015).

**FIGURE 2:**
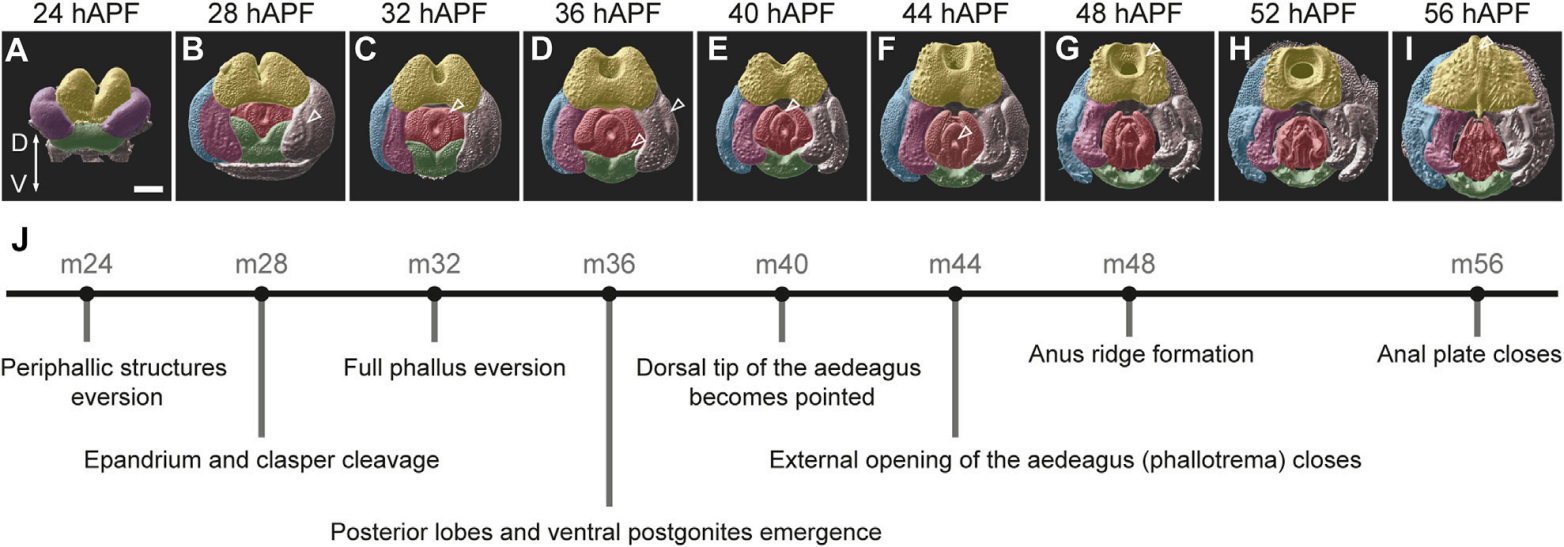
An overview of male genitalia development in *D. melanogaster*. **(A–I)** 3D surface images of male pupal terminalia from *D. melanogaster* at the indicated developmental time points. The 3D surfaces were generated from confocal images of pupal terminalia stained with anti-E-cadherin using Imaris (See Materials and Methods). False coloring marks the major substructures of the terminalia as follows: yellow—anal plate; blue—epandrium (lateral plates); pink—clasper, purple—epandrium and clasper primordium; red—phallus; and green—hypandrium. Scale bar: 50 µm. The morphological landmark described in (J) for each timepoint is marked with an arrowhead. **(J)** Suggested staging scheme. Each stage is represented by a dot positioned at the corresponding timepoint of *D. melanogaster* development, with a description of the developmental event that characterizes this stage.

All adult genital structures develop from the larval genital disc during metamorphosis. The genital disc is unique among other imaginal discs by virtue of its sexual dimorphism and its single, unpaired primordium. The male genital disc is formed by fusion of primordia originated from three embryonic abdominal segments: a reduced A8 primordium that develops into a tiny eighth tergite, and in females gives rise to most genital structures; an A9 primordium that forms the male genitalia; and the A10 primordium that produces the analia (Chen E.H. and Baker B.S., 1997; Gorfinkiel et al., 1999; Keisman and Baker, 2001; Estrada et al., 2003). During metamorphosis, the genital disc grows and remodels through extensive cell proliferation and epithelial remodeling (Estrada et al., 2003; Glassford et al., 2015; Smith et al., 2020). The major morphogenetic events that shape the genitalia in *D. melanogaster* take place between 28 h and 56 h after puparium formation (hAPF) (Glassford et al., 2015; Vincent et al., 2019). To monitor these events, we dissected and imaged the terminalia from *D. melanogaster* male pupae at 4-h intervals between 24 and 56 hAPF, stained with an anti-E-cadherin antibody that marks the apical cell junctions. We use this time series to propose a new staging scheme for male genital development that is based on characteristic developmental events during *D. melanogaster* pupal terminalia development (Figure 2). The suggested stages are named according to the time after puparium formation in which they occur in *D. melanogaster* (for example: m24—m for melanogaster and 24 for 24 hAPF, see Figure 2).

We begin our staging at 24 hAPF (stage m24), when three primordia can be distinguished externally: the dorsal anal plate primordium, the lateral epandrium and clasper primordia, and the ventral hypandrium primordium (Figure 2A). Prior to that, during the first 24 hAPF, the analia primordium everts around the posterior edge of the pupal terminalia and forms the anal tube. This event is followed by the eversion of the epandrium and clasper primordia and their positioning around the anal plate and the genital opening (Epper, 1983). At stage m24, the periphallic structures and the hypandrium are fully everted, but the phallus is internal and not easily visible from the posterior view (Figure 2A). At stage m28, the phallus everts and becomes visible between the periphallic structures. In addition, at this stage the future epandrium and clasper begin to physically separate as a cleavage appears between them (Figure 2B). By stage m32, the phallus is fully everted and both the central and lateral phallus primordia (Rice et al., 2023) become visible (Figure 2C). At stage m36 the posterior lobes become clearly distinct and the ventral postgonites of the phallus emerge (Figure 2D). At stage m40 the dorsal tip of the aedeagus changes its shape and becomes pointed (Figure 2E). Stage m44 is characterized by the closure of the phallotrema, the external opening of the aedeagus, that acquires a V shape (Figure 2F). At stage m48, all the genital substructures, including phallic substructures, are easily recognizable and a ridge-like circle forms around the anus (Figure 2G). By stage m56, the anal plates close (Figure 2I). At this stage the major morphogenetic processes that shape the external genitalia conclude and the external tissue becomes chitinized. Using these key diagnostics as a reference, we sought to test whether the same developmental timing is found in other species of the *D. melanogaster* species group.

### 3.2 A developmental atlas of pupal terminalia development across twelve species of *Drosophila*

To gain insights into the developmental processes that shape male terminalia across evolution, we expanded our developmental analysis to twelve *Drosophila* species. Our analysis includes the nine species from the *D. melanogaster* subgroup: *D. melanogaster, D. simulans, D. sechellia, D. mauritiana* of the *D. melanogaster* complex, *D. yakuba, D. santomea, D. teissieri* of the *D. yakuba* complex and *D. orena, D. erecta* of the *D. erecta* complex that radiated approximately 3.5 million years ago (MYA); one species from the *D. suzukii* subgroup (*D. biarmipes*); and two species from the *D. ananassae* subgroup (*D. malerkotliana, D. ananassae*) that diverged from the *D. melanogaster* clade 11–21 MYA (Obbard et al., 2012) (Figure 1C). We monitored pupal terminalia development for each of these species at 4-h intervals between 28 hAPF and anal plate closure (equivalent to stage m56). The full dataset is presented in Supplementary Figure S2.

We observed substantial heterochrony in pupal terminalia development between species. Nonetheless, we could align developmental timepoints across species based on the morphological characteristics that were used for the staging of pupal terminalia development in *D. melanogaster* (Figure 2; Supplementary Figure S3). This task was quite easy for species of the *D. melanogaster* complex, as they share all the temporal landmarks that exist in *D. melanogaster*. Within this group, a heterochronic shift was observed mainly for *D. simulans*, in which the terminalia develops faster than in other species of the group, making it more difficult to identify certain stages that emerge more quickly than our selected 4-h intervals (Supplementary Figure S3). Outside of the *D. melanogaster* complex, the task was more challenging as not all developmental landmarks exist, and some substructures develop at different rates in different species. However, many substructures and morphogenetic events are conserved even in distantly related species. These include lateral plate and clasper cleavage (stage m28), phallus eversion (stage m32), shape change of the dorsal tip of the aedeagus (stage m40), closure of phallotrema (stage m44), and closure of the anal plates (stage m56). These developmental landmarks allowed us to align the time series of each of the twelve species (Supplementary Figure S3).

Our dataset provides a rich ground for researchers in the field to study various aspects of genital development and evolution. Here, we highlight developmental events that lead to the formation of genital characteristics we find interesting. However, the reader is invited to carefully examine the full dataset to find their own inspiration. We have previously described the developmental processes that shape the phallic structures (Rice et al., 2023). Here, we focus on the anatomy, development, and diversification of the periphallic structures sorted by substructures.

### 3.3 The anal plates (cerci)

The anal plates (cerci) are a pair of tergites that flank the anus from both sides. They form a rather simple and conserved dome-like structure in the *D. melanogaster* complex and exhibit diverse modifications in other species (Figure 1; Supplementary Figure S1). The anal plates differ in the number and stoutness of their bristles and some species bear modified bristles that resemble teeth or spines on their ventral cercal lobes. In general, most of the modifications we observed in our analysis are on the ventral cercal lobes (also referred to as “secondary claspers”). For example, *D. teissieri* males have enlarged anal plates that harbor a set of massive teeth on their ventral lobes (Figure 1J; Supplementary Figure S1G). The ventral cercal lobes in *D. orena* expand ventrally to form large, spined extensions (Figure 1L; Supplementary Figure S1I), while the anal plates of species of the *D. ananassae* subgroup evolved sharp, sclerotized, claw-like spines (Figure 1N,O and Supplementary Figure S1K,L). It was shown that in *D. ananassae* and its close relative, *D. bipectinata*, these spines are used to grasp the female genitalia to initiate copulation, and thus are important for precopulatory sexual selection (Polak and Rashed, 2010; Grieshop and Polak, 2012; 2014). However, they also reduce the female fecundity, probably due to wounding during copulation (Grieshop and Polak, 2014; Rodriguez-Exposito et al., 2020). Similarly, it was shown that the male anal plates of various species couple with the female oviscape to facilitate genital coupling (Jagadeeshan and Singh, 2006; Kamimura and Mitsumoto, 2011; Yassin and Orgogozo, 2013).

**FIGURE 3:**
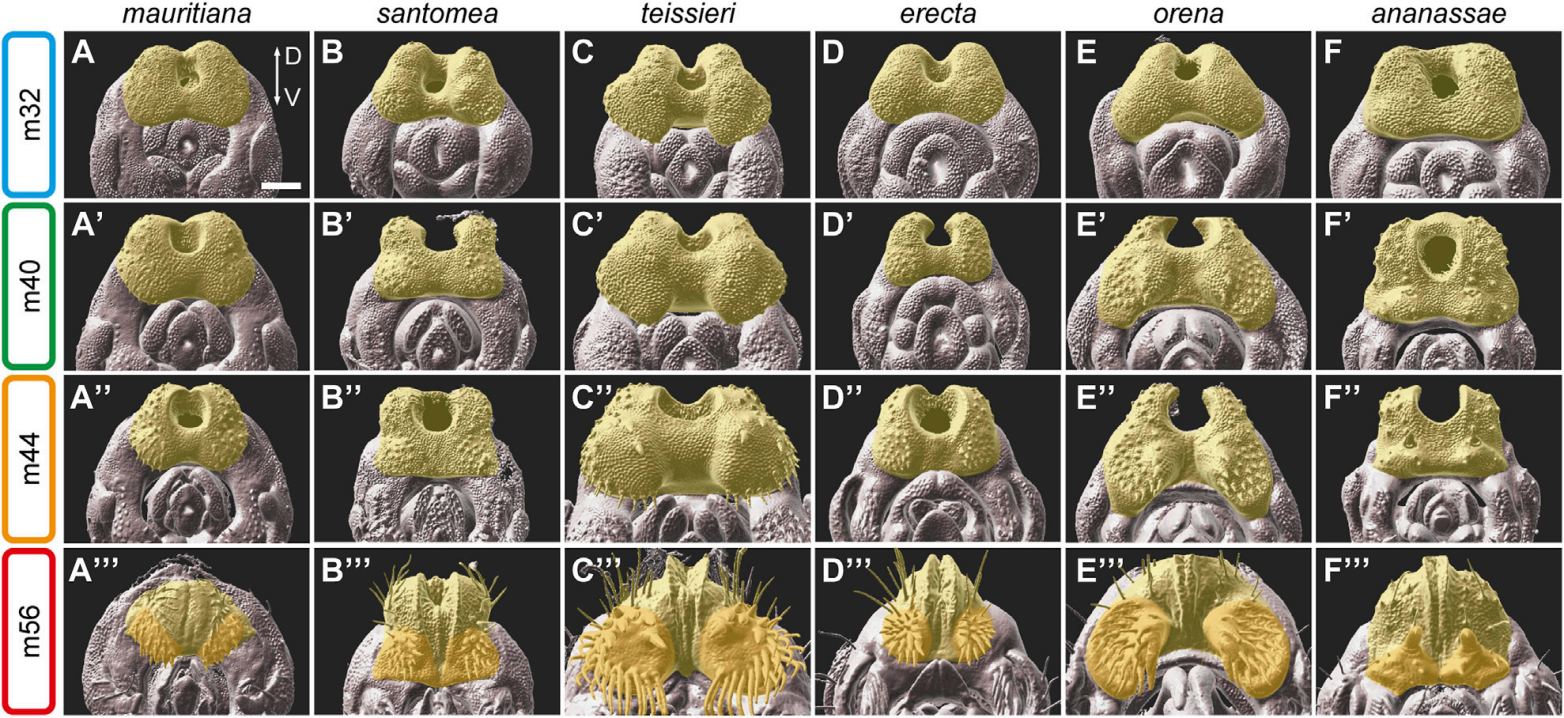
The development of the anal plate in six species of the *D. melanogaster* species group. **(A–F‴)** 3D surface images of male pupal terminalia of the species indicated on the top. The anal plate is highlighted in yellow. In stage m56 images, the ventral cercal lobe is highlighted in dark yellow. **(A–F)** Early in development, at stage m32, the anal plate morphology is relatively conserved, except for *D. teissieri* (C), which exhibits developed ventral cercal lobes. **(A′–F′)** At stage m40, differences in the shape and the size of the anal plate become clear. **(A**″**–F**″**)** At stage m44, species-specific modifications on the ventral cercal lobe, such as the outgrowths in *D. teissieri* **(C**″**)** and *D. orena* **(E**″**)** and the large pair of bristles in *D. ananassae* **(F**″**)** can be easily detected. **(A‴–F‴)** By stage m56, when the anal plates close over the gap between them, the anal plate is almost fully developed and resemble their adult shape. The modifications on the ventral cercal lobes of *D. santomea* **(B‴)**, *D. teissieri* **(C‴)**, *D. erecta* **(D‴)**, *D. orena* **(E‴)** and the spines on the ventral cercal lobes of *D. ananassae* **(F‴)** are clearly visible. Scale bar: 50 µm.

The anal primordia originate from the embryonic abdominal segment A10 in both males and females and give rise to the anal plates and the hindgut. The fate of these two territories is determined by the complementary expression of *Distal-less* and *caudal* in the analia and *even-skipped* in the hindgut (Gorfinkiel et al., 1999; Moreno and Morata, 1999). In the third-instar larval genital disc, the anal plate primordia flank the hindgut primordium on both sides (Gorfinkiel et al., 1999). During the first 24 h of metamorphosis the hindgut cells invaginate to form a tube, and the two anal plate primordia fuse to form a donut-like structure with a hole (anus) in the middle (Figure 2A). Figure 3 compares the development of the anal plates in six species that evolved specialized modifications on their anal plates, with *D. mauritiana* as a representative of the *D. melanogaster* complex.

At stage m32 of genitalia development, the anal plate morphology is quite conserved with some minor size differences between species (Figure 3; Supplementary Figure S2, *D. teissieri* and *D. orena* are an exception, see below). At this time point the anal plate bristles start to bud. Species differences in morphology become more evident at stage m36 (Supplementary Figure S2). As expected, the major species differences are observed on the ventral side of the developing anal plates. For example, in *D. teissieri*, the ventro-lateral sides of the anal plates form two enlarged cushion-like structures early on that continue to expand at later stages (Figure 3C). These structures grow two types of bristles: seven robust teeth on each dorso-medial side and around twenty finer and longer bristles on each lateral side of these extensions (Figure 3C″,3C‴). In contrast, their sibling species, *D. santomea* and *D. yakuba,* form much smaller square-shaped anal plates (Figure 3B; Supplementary Figure S2). The ventral cercal lobes of *D. santomea* and *D. yakuba* “bud” from the anal plate at late stages of pupal terminalia development (around stage m56, Figure 3B‴; Supplementary Figure S2) to form “secondary claspers” ventral to the anal plates (Supplementary Figure S1E,F). Another striking difference in the morphology of the anal plates is observed among the sibling species *D. erecta* and *D. orena*. At stage m28 they share a conserved donut-like shaped anal plate (Supplementary Figure S2), but by stage m32, the ventro-lateral sides of the *D. orena* anal plates start to expand, giving the anal plate a crescent-like shape (Figure 3E). The ventral cercal lobes of *D. orena* continue to grow to form two large processes that harbor three large spines on each medial surface and twenty thick bristles more laterally (Figure 3E‴). *D. erecta* males form significantly smaller ventral cercal lobes, but as in *D. orena*, they are covered by stout bristles (Figure 3D‴).

Our analysis also captures the development of the large spines on the ventral cercal lobes of *D. ananassae* and demonstrates that they are modified bristles. The spine buds can be first detected at stage m28 at the time the bristles start to emerge (Supplementary Figure S2). At stage m32, the spine buds look like enlarged bristle buds (Figure 3F). Next, the tissue around the buds start to condense to form a small dome (Figure 3F′). The domes and the spines continue to grow to form the “secondary claspers” and their sclerotized hooks (Figure 3F‴). A similar process is observed in males of *D. malerkotliana* that develop smaller spines on their ventral cercal lobes (Supplementary Figure S2). In *D. malerkotliana* the buds of these spines can be detected as early as 28 hAPF (Supplementary Figure S2).

Our results suggest that the anal plates are divided into two domains, a dorsal domain, that exhibits a constrained development and morphology and a ventral domain, that evolves rapidly to form specialized modifications that may facilitate species-specific coupling.

### 3.4 The claspers (surstyli) and the lateral plates (epandrial ventral lobes)

The lateral plates (epandrial ventral lobes) are a pair of protrusions that extend ventrally from the genital arch (epandrium) on opposite sides of the genitalia (Figures 1A,B). In species of the *D. melanogaster* complex, they harbor the posterior lobes that extend out of their dorsal plane posteriorly (see below). The claspers are paired sclerotized lobes that extend ventrally from the subepandrial sclerite and surround the phallus (Figures 1A,B). They vary from rather simple hook-shaped outgrowths of variable size in the *D. melanogaster* complex (Figures 1D–G; Supplementary Figures S1A–D) to robust structures in *D. teissieri* (Figure 1J; Supplementary Figure S1G) and highly complex spoon-like structures in *D. biarmipes* (Figure 1M; Supplementary Figure S1J). The claspers are characterized by species-specific arrays of stout setae that are directed medially and exhibit remarkable differences in their number, distribution, and morphology. As the name suggests, the claspers participate in clutching the female genitalia during copulation (Jagadeeshan and Singh, 2006; Kamimura and Mitsumoto, 2011; Yassin and Orgogozo, 2013). In species that lack posterior lobes, such as *D. orena* and *D. erecta*, the lateral plates participate together with the claspers and the anal plates in grasping onto the female genitalia (Yassin and Orgogozo, 2013).

**FIGURE 4:**
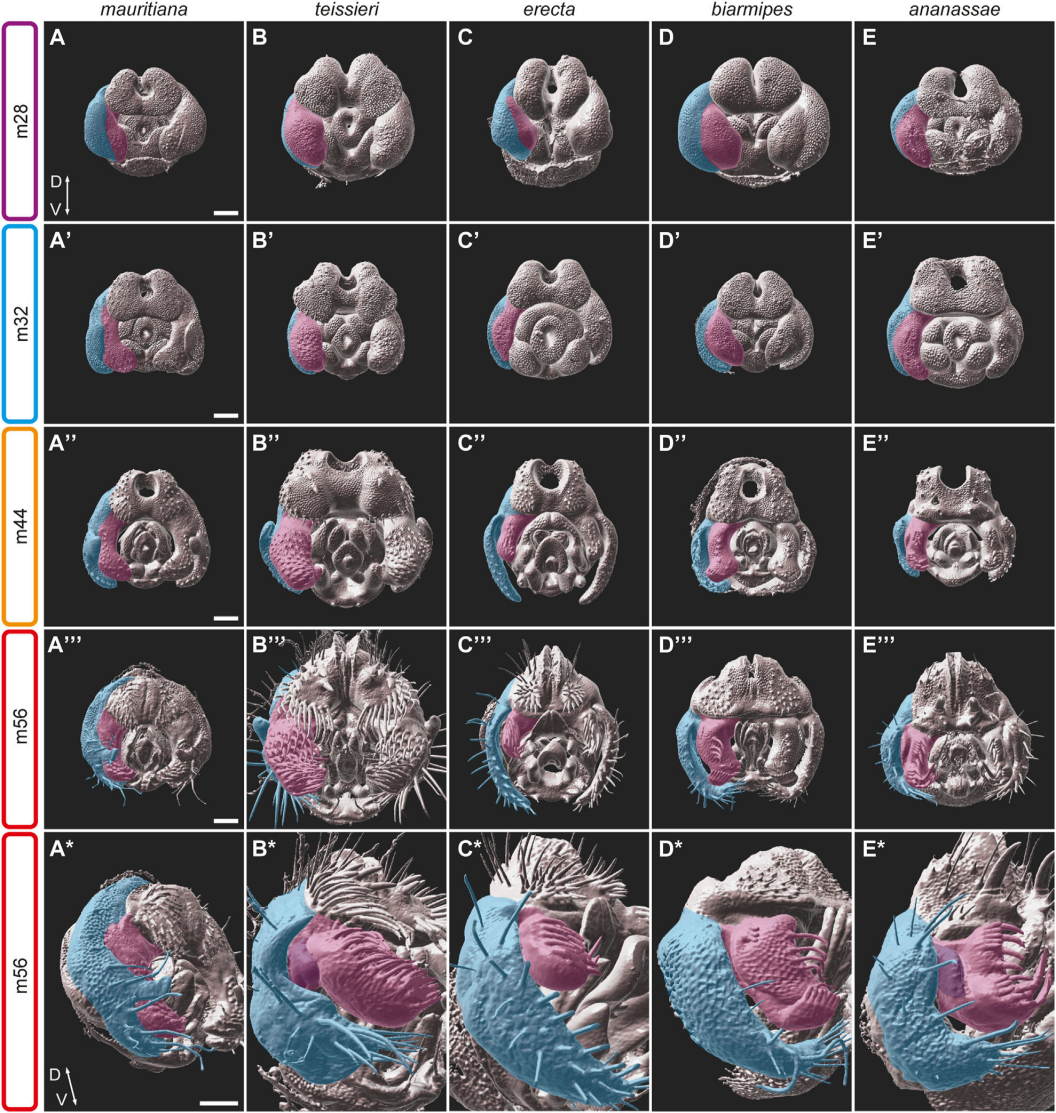
The development of the epandrium and the claspers in five species of the *D. melanogaster* species group. (A–E*) 3D surface images of male pupal terminalia of the species indicated on the top. The right epandrium and the clasper of the pupal terminalia are highlighted in blue and pink, respectively. **(A–E)** At stage m28, the epandrium and clasper primordium start to divide as a cleavage appears between the epandrium and clasper domains. **(A′–E′)** At stage m32, the epandrium and claspers continue their separation. Specific characters, like the posterior lobes on the lateral plates of *D. mauritiana* **(A′)**, the robust claspers of *D. teissieri* **(B′)** or the sex comb-like structures in *D. ananassae* **(E′)** appear. **(A**″**–E**″**)** At stage m44, the claspers display diverse morphologies including size differences (for example, *D. teissieri* **(B**″**)** and *D. erecta* **(C**″**)**, shape differences, variable outgrowth (for example, *D. biarmipes* **(D**″**)** and *D. ananassae* **(E**″**)**) and differences in bristle size, number, and patterns. **(A‴–E‴)** At stage m56, the epandrium and claspers adopt their final shape and proportions. (A*–E*) A side view of the m56 stage shows the clasper outgrowths (purple) in *D. teissieri* (B*) and *D. ananassae* (E*) and the unique bristle pattern in *D. biarmipes* (D*). Scale bars: 50 µm.

The lateral plates and the claspers develop from shared primordia that originate from abdominal segment A9. During stage m24, the primordia can be seen flanking the anal plate primordium on both sides (Figure 2A). By stage m28 the lateral plate and the clasper begin their physical separation as a cleavage forms between the two territories (Figure 2B; Supplementary Figure S2). The clasper territory can be distinguished prior to the physical separation from the lateral plate by the expression of *odd paired* (*opa*), while *empty spiracles* (*ems*) marks the position of the cleavage (Vincent et al., 2019). The location of the cleavage between the lateral plate and the clasper may influence the relative sizes of the adult structures and may represent a tradeoff in resource allocation. For example, in species of the *D. melanogaster* complex that develop enlarged posterior lobes on their lateral plates, the lateral plate territory seems to be relatively large (Figure 4A; Supplementary Figure S2). A similar trend is observed in *D. erecta* which possesses extended lateral plates and short claspers (Figure 1K; Figure 4C). In contrast, in *D. teissieri,* their robust clasper territory expands to the seeming expense of the lateral plate (Figure 1J; Figure 4B). Besides the differences in territory sizes, the morphologies of the claspers and lateral plates at early developmental stages (i.e., stage m32 and earlier) are quite conserved (Figure 4; Supplementary Figure S2). Following the separation from the lateral plates (at around stage m36), the claspers form similar rounded elliptical structures in which the future medial surfaces face posteriorly. This surface carries species-specific arrays of bristles that can be first detected even prior to the separation from the lateral plates (Figure 4; Supplementary Figure S2). As development proceeds, the claspers take their final shape while condensing and rotating medially, so that the bristle arrays face medially.

As noted above, species differences in clasper morphology include differences in size and shape, as well as bristle number and morphology. Size differences can be seen even between closely related species. For example, species of the *D. melanogaster* complex share similar clasper morphogenesis but differ in clasper size and in the number and stoutness of the bristles they carry. The size differences can be detected from the initiation of clasper development, where *D. mauritiana* males form broad claspers and *D. simulans* form narrow ones (Figure 4A; Supplementary Figure S2). A parallel trend is observed in *D. santomea* and *D. yakuba* which share similar clasper shape but differ in size: *D. yakuba* develop significantly smaller claspers compared to *D. santomea* (Supplementary Figure S2). In contrast, the third member of the *D. yakuba* complex, *D. teissieri*, forms enlarged claspers that are covered by dozens of stout bristles (Figure 4B). *D. teissieri* also contains a morphology not found in any other species analyzed in this study. The ventral medial portion of the clasper of *D. teissieri* houses a small finger-like extension that is somewhat obscured by the many bristles that cover the clasper and can be best seen at stage m44, when the bristles are still in the process of extending (Supplementary Figure S1G; Figure 4B″). Additionally, we find that *D. biarmipes* has evolved a lobe-shaped extension in the ventral lateral region of the clasper, which develops at stage m40 and houses a row of darkly pigmented bristles (Supplementary Figure S1J; Figure 4D*). We did not observe any outgrowths or modifications in the ventral lateral region of the clasper in any other species analyzed.

In general, the claspers of all the species in our dataset are decorated with many sensory bristles with varied sizes and shapes. The number of bristles varies substantially, from 8 bristles on the medial surface of the claspers of *D. biarmipes* to the 56 bristles that cover the broad claspers of *D. teissieri*. These bristles start to extend out from the surface between stages m28-36. In addition to these sensory bristles, all species we analyzed contain thick darkly pigmented bristles on their claspers (Supplementary Figure S1). These structures were previously described as bristles or teeth in different species from several subgroups, including *D. biarmipes*, *D. suzukii*, *D. takahashii* (Kopp and True, 2002). Interestingly, these clasper bristles, especially those found in *D. biarmipes*, *D. ananassae,* and *D. malerkotliana* (Supplementary Figure S1J–L), resemble the sex combs that characterize the first pair of legs in males of the *D. melanogaster* and *D. pseudoobscura* species groups. While the shape, the number of teeth and the location of the two sets of sex comb-like structures vary between species, the striking similarity in the “teeth” morphology suggests that these structures are homologous. Our confocal images demonstrate that these structures indeed, develop in a comparable way regardless of their exact position within the clasper (Figures 4D,E; Supplementary Figure S2).

Finally, our 3D confocal images have revealed uncharacterized outgrowths on the dorso-lateral side of the claspers of *D. teissieri, D. ananassae*, and *D. malerkotliana* (Figure 4B*, 4E*; Supplementary Figure S2). These outgrowths were first observed at relatively late stages of pupal terminalia development (48, 44 and 40 hAPF, respectively) and they develop into a fold on the lateral side of the claspers (Figures 1J,N,O). While *D. ananassae* and *D. malerkotliana* both represent the *D. ananassae* species subgroup and have similar morphology, *D. teissieri* belongs to the *D. melanogaster* subgroup, which is fully represented in the current study and where no such clasper outgrowths were observed. Considering the phylogenetic relations of *D. ananassae* and *D. malerkotliana* and the similarities in their clasper development, these substructures seem to be homologous in these two species. However, the clasper outgrowth in *D. teissieri* is unique within the *D. melanogaster* subgroup and likely evolved independently.

### 3.5 The posterior lobes (epandrial posterior lobes)

The most dramatic differences in genitalia morphology among species of the *D. melanogaster* complex is in the shape and size of the posterior lobes (Coyne, 1983; Jagadeeshan and Singh, 2006; Yassin and Orgogozo, 2013). The posterior lobes protrude from the lateral plates and are used for grasping the female genitalia during copulation (Kamimura and Mitsumoto, 2011; Yassin and Orgogozo, 2013). They vary from small “hook-like” projections in *D. melanogaster* (Figure 1D) to elaborated “clamshell” shape in *D. simulans* (Figure 1E; Sturtevant, 1919) and “finger-like” structures in *D. mauritiana* (Figure 1G)*.* They have been the subject of numerous evolutionary, functional, genetic, and developmental studies and are considered an evolutionary innovation in the *D. melanogaster* complex (Coyne, 1983; Masly et al., 2011; Frazee and Masly, 2015; Glassford et al., 2015; Smith et al., 2020; Frazee et al., 2021; Ridgway et al., 2023). Nonetheless, species of the *D. yakuba* complex also exhibit small projections on their lateral plates that might be homologous to the posterior lobes (Figures 1H–J; Supplementary Figures S1E–G) (Jagadeeshan and Singh, 2006; Yassin and Orgogozo, 2013).

**FIGURE 5:**
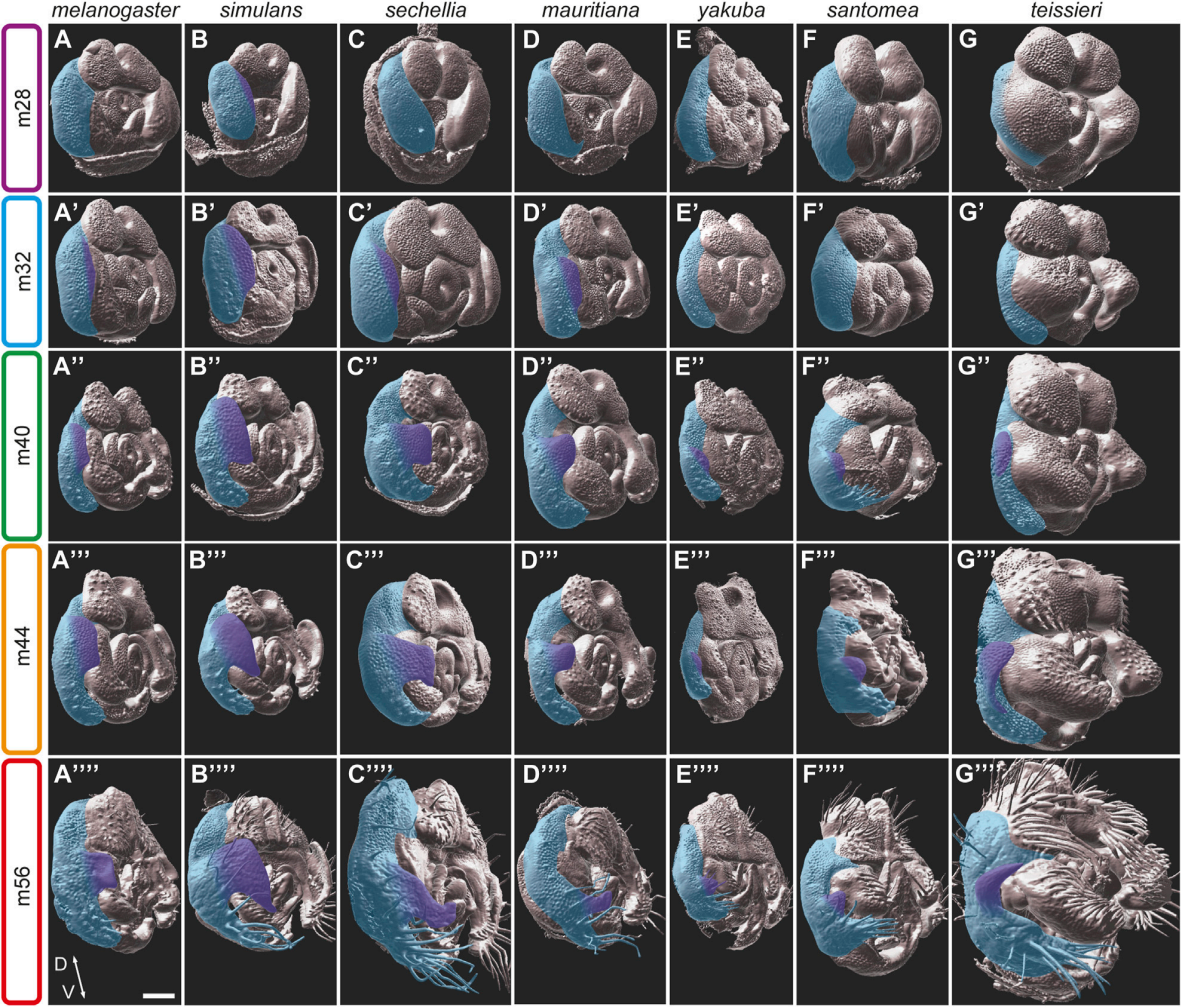
The development of the posterior lobes in the *D. melanogaster* subgroup. (A–G*) 3D surface side views of male pupal terminalia of the species indicated on the top. The right epandrium and posterior lobe of the pupal terminalia are highlighted in blue and purple, respectively. **(A–G)** At an early developmental stage m28, *D. simulans* **(B)** is the only species that shows initiation of posterior lobe growth. **(A′–G′)** At stage m32, the posterior lobe initiation appears in *D. melanogaster* **(A′)**, *D. sechellia* **(C′)** and *D. mauritiana* **(D′)**. **(A**″**–G**″**)** At stage m40, the posterior lobes of the *D. melanogaster* complex **(A**″**–D**″**)** continue to grow and shape, as the posterior lobes of the *D. yakuba* **(E**″**)**, *D. santomea* **(F**″**)**, and *D. teissieri* **(G**″**)** begin to protrude from their lateral plates. **(A‴–G‴)** At stage m44, the posterior lobe continues to grow, and begins to shrink at the dorso-ventral axis to acquire it specific shape in *D. melanogaster* **(A‴)**, *D. simulans* **(B‴)**, *D. sechellia* **(C‴)**, and *D. mauritiana* **(D‴)**. **(A‴‘–G‴‘)** At stage m56, the posterior lobes acquire their final shapes. Scale bar: 50 µm.

Smith et al. (2020) have recently provided a detailed analysis of the *D. melanogaster* posterior lobe morphogenesis*.* They revealed that the posterior lobes start to emerge from the lateral plates at stage m36 following the separation between the lateral plates and the claspers. The posterior lobes then extend to their final shape through apico-basal cell elongation facilitated by interactions with the aECM protein Dumpy (Smith et al., 2020). Most of this elongation takes place at the final steps of posterior lobe morphogenesis between 48 and 52 hAPF, in which the posterior lobes double their height. Our analysis reveals that *D. sechellia* and *D. mauritiana* posterior lobes follow a similar developmental timeline as the *D. melanogaster* posterior lobes. In both species, the posterior lobes protrude from the lateral plates at a more ventral position compared to *D. melanogaster* (compare Figures 5A′, 5C). In *D. sechellia,* a broader field of cells projects out of the surrounding epithelium early on, and the posterior lobes elongate faster and further compared to those of *D. melanogaster* and *D. mauritiana*. As the *D. sechellia* posterior lobes develop they narrow to form long, thin, and flat structures (Figure 5C′–C‴′). The posterior lobes of *D. mauritiana* develop from a comparably sized cell primordium as in *D. sechellia* (Figure 5D′). They however acquire their “finger-like” shape through extensive elongation and narrowing, similar to the *D. sechellia* posterior lobes (Figure 5D″–D‴′).

Among the species of the *D. melanogaster* complex, *D. simulans* stands out due to its distinctive and elaborated posterior lobes. The development of their large “clamshell” shaped posterior lobes exhibits both heterochronic and morphogenetic differences when compared to other species in the group. The *D. simulans* lateral plates start to separate from the claspers prior to 28 hAPF. By stage m28, the *D. simulans* posterior lobes are already apparent (Figure 5B). The field of cells that project to form the posterior lobes extend from the dorsal part of the lateral plates ventrally to encompass almost two-thirds of the medial lateral plates (Figure 5B′). The posterior lobes continue to grow while adopting their characteristic shape by stage m40 (Figure 5B″), and soon after, they acquire their final shape and size. Future analyses will determine what kind of cell behaviour(s) participate in the shaping of these structures.

While species of the *D. melanogaster* complex possess distinct posterior lobes, some species of the *D. yakuba* complex have small processes that extend from the apical ends of the lateral plates. These processes vary from very small extensions in *D. yakuba* (Figure 1H), to larger extensions in *D. santomea* (Figure 1I), to enlarged spikes in *D. teissieri* (Figure 1J). These processes start to emerge from the lateral plates relatively late at stage m40, compared with the posterior lobes of species in the *D. melanogaster* complex. In addition, they form at a more ventral position relative to the posterior lobes of the *D. melanogaster* complex from much smaller cell primordia. Nonetheless, the morphogenesis of these processes closely resembles the developmental events shaping the posterior lobes of the *D. melanogaster* complex, suggesting that they are homologs.

### 3.6 The emergence of the posterior lobe preceded the split between the *D. melanogaster* and the *D. yakuba* complexes

**FIGURE 6:**
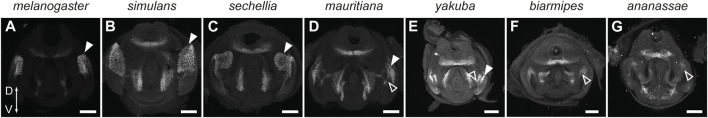
Ems marks the posterior lobe in the *D. melanogaster* subgroup of species. **(A–G)** Confocal images of 48 hAPF pupal terminalia dissected from the lobed species *D. melanogaster* **(A)** *, D. simulans* **(B)** *, D. sechellia* **(C)** *, D. mauritiana* **(D)** *,* and *D. yakuba* **(E)** and the non-lobed species, *D. biarmipes* **(F)** and *D. ananassae* **(G)**, immunostained with anti-Ems antibodies. White arrowheads indicate the expression associated with the posterior lobes. Empty arrowheads show the first wave of Ems expression at the border between the lateral plates and claspers. Scale bars: 50 µm.

Our developmental analyses suggest that the small processes in the *D. yakuba* complex are homologous to the enlarged posterior lobes observed in the *D. melanogaster* complex. Another way to ascertain homology is by looking at shared genetic signatures in the homologous structures. The posterior lobe emerged in part through the co-option of an *Abdominal-B* (*Abd-B*) and *Pox-neuro* (*Poxn*)-regulated network that ancestrally controls the formation of the larval posterior spiracles during embryogenesis (Glassford et al., 2015). One of the downstream targets of this network is the *ems* gene that encodes a homeodomain transcription factor involved in spiracle morphogenesis and posterior lobe formation. Ems is expressed in two waves during genitalia development. In the first wave it is expressed in the cleavage between the lateral plate and clasper, prior to posterior lobe emergence in both lobed and non-lobed species such as *D. biarmipes* and *D. ananassae* (Glassford et al., 2015). In the second wave, it is expressed in the developing posterior lobe of *D. melanogaster* (Glassford et al., 2015). We therefore used Ems as a marker for the posterior lobe fate. Ems exhibits strong expression in the posterior lobes of all four species from the *D. melanogaster* complex (Figures 6A–D). On the other hand, *D. biarmipes* and *D. ananassae* show only faint expression of Ems in the dorso-medial side of the lateral plates that represents the first wave of Ems expression (Figures 6F,G). Interestingly, in *D. yakuba,* Ems is strongly expressed in the small processes that protrude from the lateral plates. Thus, molecularly, the small protrusions observed on the lateral plates of *D. yakuba* seem to be homologous to the posterior lobes of the *D. melanogaster* complex. These results suggest that a small posterior lobe emerged in the *D. melanogaster* group before the split between the *D. melanogaster* and *D. yakuba* complexes. It is possible that the absence of projections on the lateral plates of *D. erecta* resulted from a subsequent loss as observed for other morphological traits (Stern and Frankel, 2013; Ling et al., 2023). Future work investigating the expression and the regulatory sequences of the posterior lobe network within the *D. melanogaster* group will be necessary to distinguish between repeated loss or repeated gain.

## 4 Discussion

Reproductive structures are amongst the most rapidly evolving anatomical features in the animal kingdom. Here, we have described the developmental trajectories of terminalia across a wide range of species that include the well-studied model organism *D. melanogaster.* Doing so with high resolution three-dimensional confocal imaging has revealed a treasure trove of novel processes and hidden homology relationships between structures that would otherwise appear to have evolved independently. Our results highlight how novel traits may arise from barely recognizable rudiments that can only be visualized through a careful analysis of tissue formation in a comparative framework. Below, we discuss approaches to further trace the evolutionary history of these structures at the molecular level. The seemingly endless diversity of genital structures implies that many new morphogenetic processes await discovery in these systems.

Our comparative developmental analyses permitted the discovery of previously undefined structures and allowed us to trace their cellular origins. For example, we identified uncharacterized outgrowths on the border between the lateral plates and the claspers that develop into a fold on the lateral side of the claspers in *D. teissieri, D. ananassae*, and *D. malerkotliana*. These outgrowths may have evolved through convergence, differential retention, or a cryptic atavism that reactivates an ancestral potential. In addition, we detected a small finger-like extension on the ventral medial portion of the clasper of *D. teissieri*. These structures likely went unnoticed due to the two-dimensional nature of taxonomic descriptions in past decades. Key taxonomic texts used two-dimensional camera lucida drawings based upon flattened adult cuticle preparations visualized by brightfield microscopy (e.g., Bock and Wheeler, 1972). Scanning EM micrographs of many of these species have been published, but subsuperficial structures are often obscured and are difficult to resolve. Three-dimensional confocal images offer several advantages: 1) the imaging can often resolve structures that are obscured by other structures; 2) using advanced imaging software (e.g., Imaris or morphographX), the resulting three-dimensional images can be rotated and resliced to examine particular substructures during a developmental trajectory; 3) developmental time courses can capture the formation and regression of substructures, providing a more accurate phylogenetic interpretation of homology relationships. While it is not clear if these substructures have function, their presence highlights the remarkable plasticity of genitalia primordia and their tendency to activate new developmental programs to allow rapid diversification.

Our analyses also help to distinguish the origin of substructures that were previously associated with another structure. The male genitalia in the *D. ananassae* complex bear structures known as secondary claspers. Our developmental analysis identifies that these are, in fact, extensions of the ventral cercal lobes of the anal plates, as had been hypothesized in previous studies (Polak and Rashed, 2010; Kamimura and Polak, 2011). While the ventral anal plates exhibit such diversity, the dorsal cercal lobes of the anal plates are quite conserved. During development, a division is formed between the dorsal and ventral portions of the anal plate that essentially separates the anal plate into two distinct segments. We predict that this division will also be reflected molecularly. Although our previous studies (Vincent et al., 2019) did not find transcription factors that clearly delaminate the uniform *D. melanogaster* anal plate into dorsal and ventral sections, we predict that species with distinct ventral morphologies have evolved ventral-specific regulatory factors. Interestingly, Ems, which is presented in this study as a marker for posterior lobe development, is also expressed in the ventral anal plates in all the species we analyzed, including *D. ananassae* that form “secondary claspers” (Figure 6). Further study will determine the relevance of Ems expression to the morphogenesis of the ventral cercal lobes.

One of the most diverse characteristics of genital structures are the bristles that decorate them. We see the gain and loss of large, heavily pigmented bristles across the anal plates and the claspers. All species analyzed in this study show this morphology in at least a subset of the bristles within the clasper. *D. mauritiana*, and all members of the *D. yakuba* and *D. erecta* complexes also contain bristles with a tooth-like morphology in the ventral anal plate. The gain of the tooth-like morphology in the anal plate may have been caused by the expansion of the clasper tooth genetic network to the neighboring anal plate. This tooth morphology is also shared with another well-studied bristle, that of the sex comb of the male foreleg. It has been posited that the sex comb may have co-opted the genetic network needed for this morphology from the bristles of the terminalia (Kopp, 2011). One candidate gene for this co-option event is the transcription factor *doublesex,* which is known to control the sex comb morphology in the leg and is expressed in the *D. melanogaster* clasper teeth as well (Robinett et al., 2010; Tanaka et al., 2011; Rice et al., 2019b).

So far, the genes and GRNs that participate in terminalia morphogenesis and diversifications have been studied almost exclusively in the context of *D. melanogaster* and its sibling species. While the powerful genetic toolkit of *D. melanogaster* allows interrogating these pathways at high resolution, working in species outside *D. melanogaster* is both necessary and more challenging. Focusing on too few species may overlook more complex ancestral processes that have been simplified in the focal species (Church and Extavour, 2020; Rice et al., 2023). Studying the developmental events that shape the structures we traced in the current study at the molecular level will require new experimental strategies. In this aspect, single-cell genomics and CRISPR/Cas9-mediated genome editing provide a promising avenue. Single-cell RNA sequencing holds the potential to access transcriptomes of cells in specific substructures of the pupal terminalia and to compare them across species. Our developmental time course can be used to choose the appropriate developmental timepoints for such analysis. Such experiments could, for example, differentiate molecularly between the dorsal and the ventral segments of the anal plates in species with modified ventral cercal lobes. Additionally, they may reveal shared ventral genetic signatures among these species. Subsequently, CRISPR/Cas9-mediated genome editing can be used for functional validation of potential regulators identified in single-cell experiments.

## Data Availability

The original contributions presented in the study are included in the article/Supplementary Materials, further inquiries can be directed to the corresponding authors.
