# Supplementary material for: A developmental atlas of male terminalia across twelve species of Drosophila
Source: Front Cell Dev Biol. 2024 Feb 29;12:1349275. doi: 10.3389/fcell.2024.1349275 (PMC10937369; doi:10.3389/fcell.2024.1349275)
Supplement: Supplementary file 2 [file Table1.DOCX]

Supplementary Material

## Supplementary Tables

**Table S1: The source of confocal images used in this paper.**

The table indicates the source of confocal files presented in this study. Original confocal files from this paper are indicated in light gray. Confocal files that were previously used in (Rice et al., 2023) are indicated in dark gray.

## Supplementary Figures

**Figure S1: Adult periphallic structures of twelve species of the *D.* *melanogaster* species group.**

(A-L) Light microscopy images of adult periphallic structures (the phallus and hypandrium were removed) of *D. melanogaster* (A), *D. simulans* (B), *D. sechellia* (C), *D. mauritiana* (D), *D. yakuba* (E), *D. santomea* (F), *D. teissieri* (G), *D. erecta* (F), *D. orena* (I), *D. biarmipes* (J), *D. malerkotliana* (K), and *D. ananassae* (L). Empty arrowhead in (G) indicates the finger-like outgrowth on the claspers of *D. teissieri*. Black arrowheads indicate the sex comb-like bristles on the claspers of *D. biarmipes* (J), *D. malerkotliana* (K) and *D. ananassae* (L). Dorso-Ventral (D-V) axis direction is indicated in panel (A). Scale bar: 50 µm.

**Figure S2: Full time course of genital disc development across the *D.* *melanogaster* species group.**

3D surface images of male genital discs from pupae of the species indicated on the right. The 3D surfaces were generated from confocal images of genital discs stained with anti-E-cadherin using Imaris (See Materials and Methods). The developmental timepoints in which the discs were dissected are indicated at the top. Colored frames mark timepoints that correspond to specific stages in the *D. melanogaster* staging scheme. Color-coding is as follows: purple - stage m28, blue - stage m32, green - stage m40, orange - stage m44, red - stage m56. D-V axis direction is indicated in the top left panel. Scale bar: 50 µm.

**Figure S3: A summary of our designations of developmental timepoints corresponding to the *D. melanogaster* staging mechanism for each species.** Stage m28 was designated when a cleavage between the lateral plate and clasper first forms. Stage m32 was designated when the phallus fully everts. Stage m40 was designated when the dorsal tip of the aedeagus changes its shape and becomes more pointed. Stage m44 was designated when the phalloterma closes. Stage m56 was designated when the anal plates close over the gap between them.
