# Supplementary figures and images for: A developmental atlas of male terminalia across twelve species of Drosophila
Source: Front Cell Dev Biol. 2024 Feb 29;12:1349275. doi: 10.3389/fcell.2024.1349275 (PMC10937369; doi:10.3389/fcell.2024.1349275)

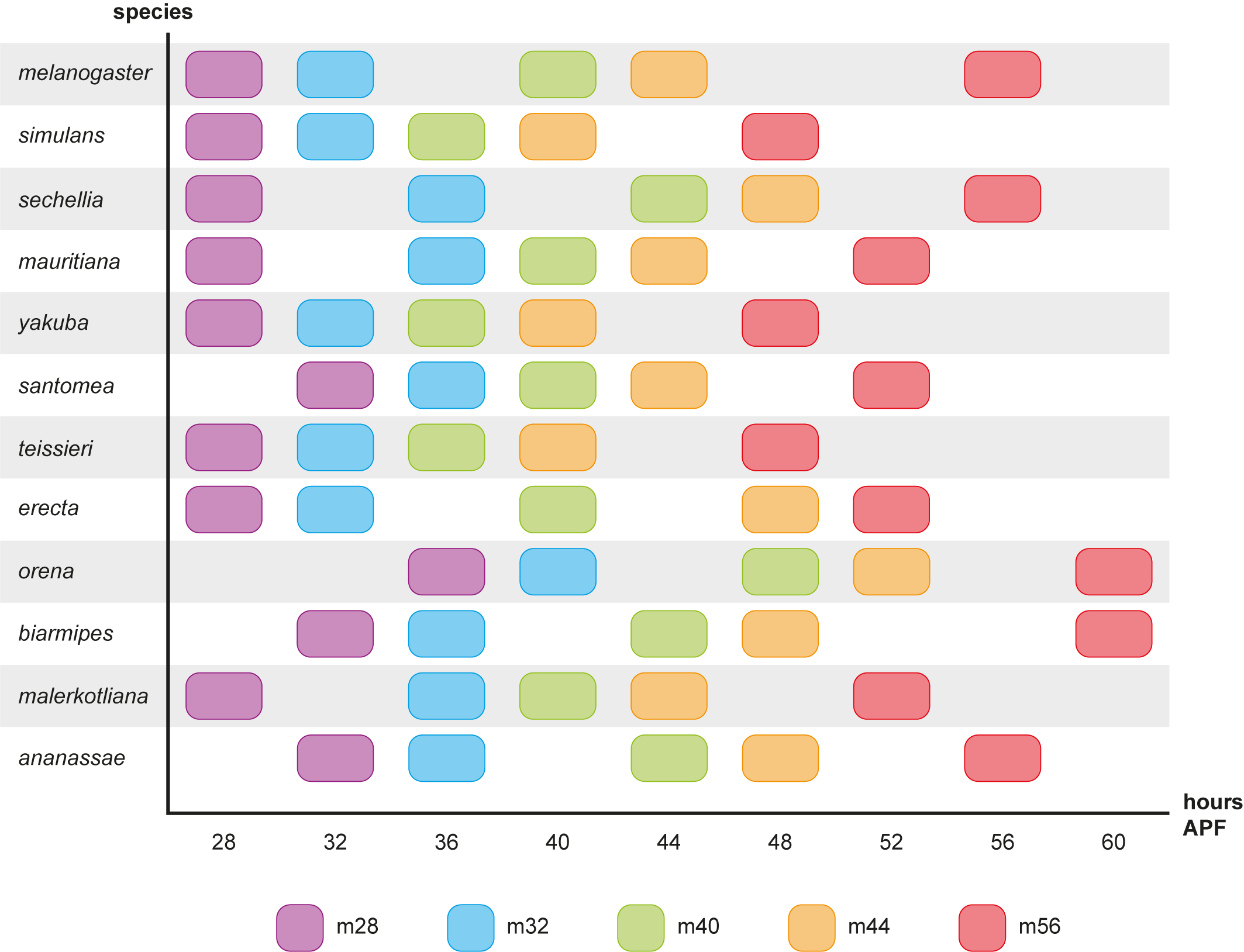

Supplement: Supplementary file 3 [file Image3.TIF]

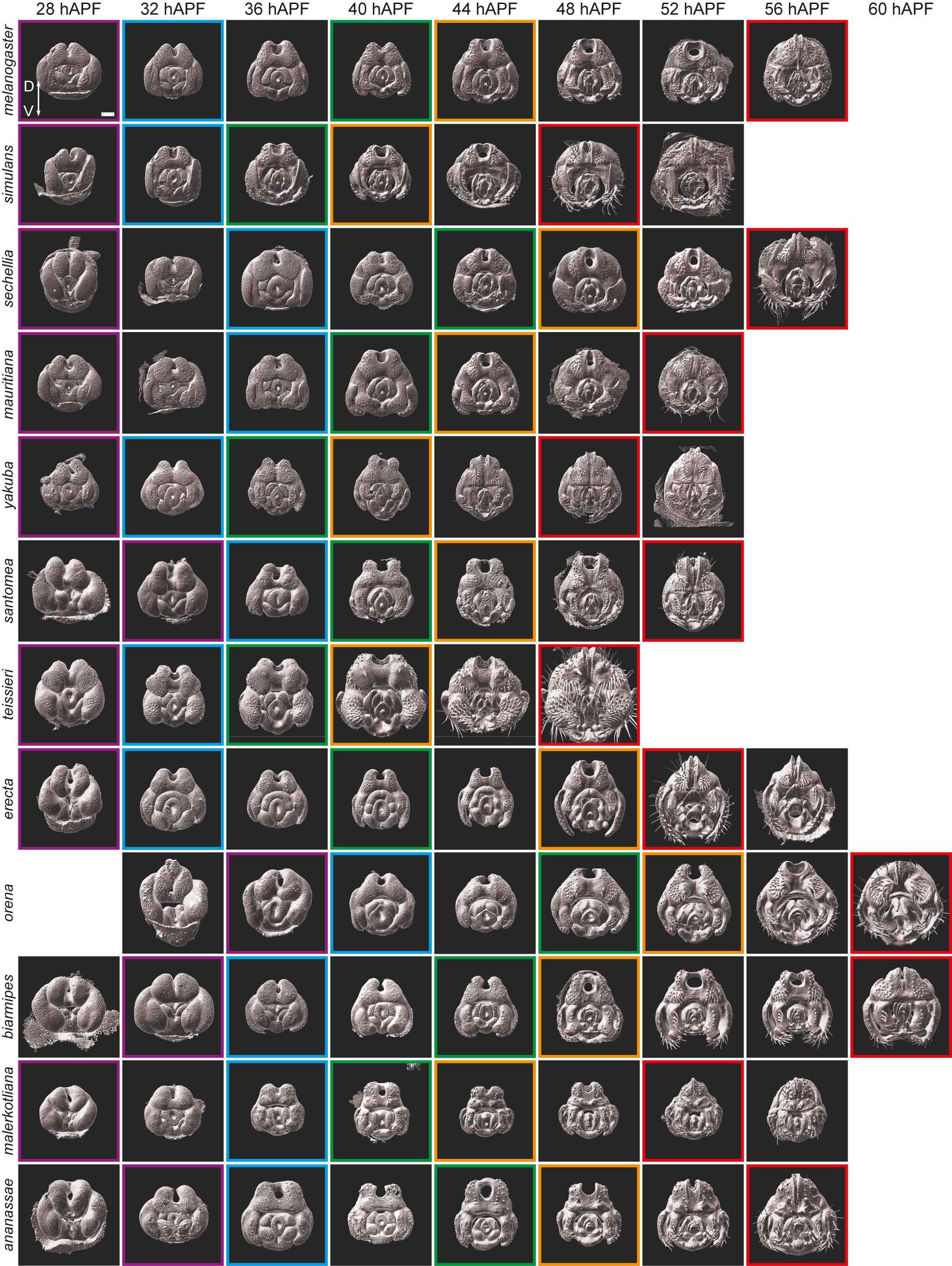

Supplement: Supplementary file 4 [file Image2.TIF]

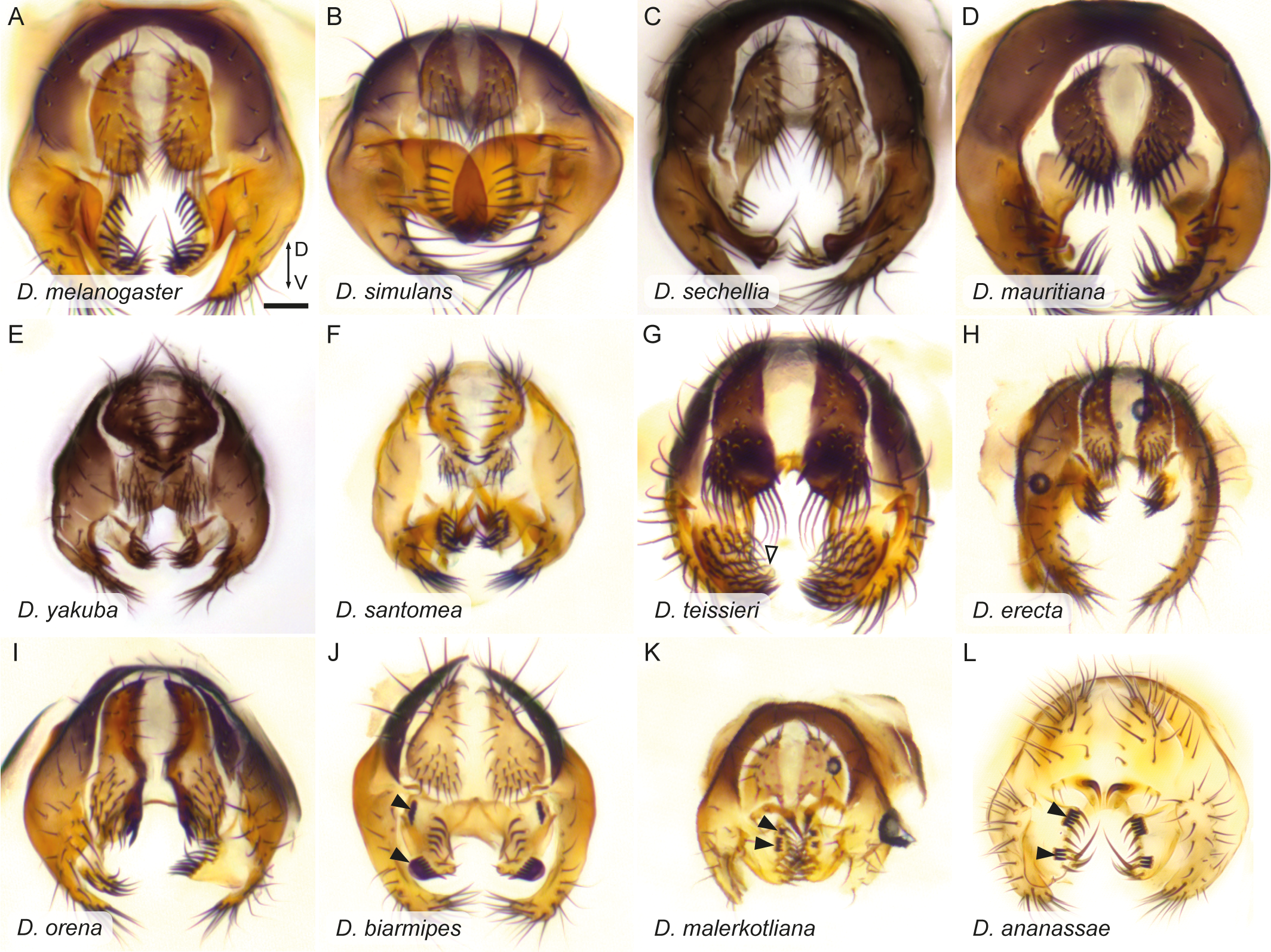

Supplement: Supplementary file 5 [file Image1.TIF]
